# Ethan D. Bolker und Maura B. Mast: Common Sense Mathematics, Second Edition: AMS/MAA Press 2021, XX + 342 Seiten, ISBN: 978-1-4704-6134-8, €73,11; e-Book ISBN 978-1-4704-6289-5

**DOI:** 10.1007/s00591-021-00314-7

**Published:** 2021-12-22

**Authors:** Joachim Hilgert

**Affiliations:** Universität Paderborn, Paderborn, Deutschland


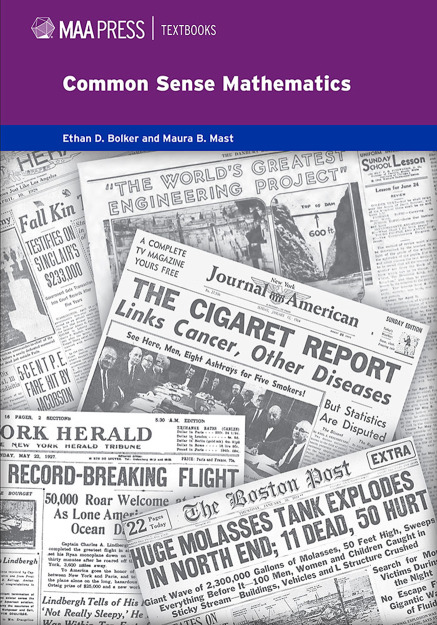


Bei vielen der gegenwärtig in unserer Gesellschaft diskutierten Themen ist man ohne ein Mindestmaß an mathematischem Verständnis bei der Meinungsbildung vollständig darauf angewiesen, Einschätzungen von vertrauenswürdigen Quellen zu übernehmen. Diese zu identifizieren ist nicht leicht, und auch vertrauenswürdige Quellen unterscheiden sich in ihren Einschätzungen. Das hier zu besprechende Buch soll und kann einen kleinen Beitrag zur Stärkung der intellektuellen Autonomie von Nicht-Experten leisten. Thema ist der Umgang mit Zahlen, die zur Beschreibung und Beurteilung realer Vorgänge eingesetzt werden.

*Common Sense Mathematics* ist ein Lehrbuch, das an amerikanischen Colleges verwendet wird und sich nicht an zukünftige MINT-Profis richtet, sondern an alle, die irgendeine Form von Hochschulabschluss anstreben. Es setzt auch ganz profan bei Alltagsfragen an, wie dem Verständnis der eigenen Stromrechnung, der Rückzahlung von Immobiliendarlehen, der Steuerprogression oder der Bestimmung der Inflationsrate. Die Mathematik, die in solchen Kontexten erklärt wird, geht eigentlich nicht über den Stoff der Sekundarstufe an deutschen allgemeinbildenden Schulen hinaus: Bruchrechnen, Rechnen mit Einheiten, Prozentrechnen, Zins und Zinseszins. Dazu kommen einfachste funktionale Zusammenhänge, ein wenig kombinatorische Wahrscheinlichkeitsrechnung, graphische Darstellungen und der Einsatz von Spreadsheets wie Microsoft Excel. Damit gelingt es den Autoren dennoch, auch etwas über Klimamodelle, exponentielles Wachstum, die Qualitätsbeurteilung von Tests oder das Framing von Einkommensverteilungen zu sagen, Themen also, die deutlich über elementare Finanzmathematik hinaus reichen.

Die Autoren gehen bei allen ihren Erklärungen von konkreten Beispielen aus und streben keinerlei Theoriebildung an. Sie nennen aber zu Beginn jeden Kapitels etliche Lernziele. Großen Wert legen sie insbesondere auf aktives Einüben des Stoffs. Zu jedem der 13 Kapitel gibt es eine umfangreiche Sammlung von Übungsaufgaben, auf der Verlags-Homepage findet man unter http://www.ams.org/publications/authors/books/postpub/text-63-extra-exercises.pdf weitere 100 Seiten mit Übungsaufgaben. Viele der Aufgaben enthalten einen expliziten Hinweis darauf, welche Lernziele mit der jeweiligen Aufgabe angesprochen werden sollen. Nicht selten geht es bei den Aufgaben darum, einen kurzen Abschnitt aus einem Zeitungsartikel zu analysieren, die Fakten zu checken und die den angegebenen Zahlen zugrundeliegenden Rechnungen durchzuführen. Wer die Aufgaben dieses Buches durchgearbeitet hat, wird sich von Inzidenzzahlen, Impfquoten, Nominal- und effektiven Zinssätzen nicht mehr so schnell einschüchtern lassen und auch von großen Zahlen nicht übermässig beeindruckt sein.

Es geht in diesem Buchen um konkretes Rechnen, wobei nicht einfach auswendig gelernte Algorithmen abgespult werden, sondern der Weg von den verfügbaren Informationen zu einem vernünftigen Ergebnis am Beispiel vorgeführt und in den Übung eigenständig erforscht wird. Zunächst geht es um grobe Überschlagsrechnungen, die dazu da sind, die Sinnhaftigkeit von behaupteten numerischen Fakten zu hinterfragen. Auch die Frage nach den Einheiten, die zusammenpassen müssen, wenn die Rechnung sinnvoll sein soll, spielt eine Rolle. Die so geübten Prinzipien bleiben den ganzen Text über in Gebrauch, auch wenn es später um das Auf- und Abzinsen oder um unterschiedliche Durchschnittsbildungen geht. Digitale Hilfsmittel werden propagiert, wo sie einen nachvollziehbaren Mehrwert liefern (der dann auch thematisiert wird), und ihr Einsatz anhand von Aufgaben geübt. Begrifflich sind elementare Konzepte der kombinatorischen Wahrscheinlichkeitsrechnung der Höhepunkt des Buches.

Zugegebenermaßen fühlte ich mich beim Lesen immer wieder an meine Schulzeit, speziell den Mathematik- und Physikunterricht in der Mittelstufe, erinnert. Dort sind mir die meisten Methoden, Tipps und Tricks, die Bolker und Mast erläutern, auch schon begegnet. Noch vor zwanzig Jahren hätte ich in einer Besprechung wahrscheinlich gefragt, ob man das Thema in Deutschland nicht eher für Schulen anstatt für Hochschulen aufbereiten sollte. Aber die gerade im Kontext der Covid-19 Pandemie offenbar gewordenen Verständnisschwierigkeiten für Zahlenzusammenhänge auch in gebildeten Schichten zeigt, dass ein solches Buch auch bei uns, trotz (oder vielleicht auch wegen) der Vielzahl „progressiver“ Reformen der letzten Dekaden im Unterrichtswesen, nicht überflüssig ist. Als wirklich erfrischend empfand ich den Mut der Autoren, sich mit allereinfachster Mathematik zu begnügen, dafür aber fast ausschließlich mit Originaldaten zu arbeiten und komplett auf artifizielle Beispiele „anwendungsnaher“ Mathematik zu verzichten. Welch ein Kontrast zu den omnipräsenten kubischen Polynomen, die in deutschen Abituraufgaben die krudesten Dinge modellieren und dann doch nur Anlass für eine immer gleiche Kurvendiskussion ohne jeden Erkenntniswert sind!

Die Beispiele und Übungsaufgaben speisen sich aus der Erfahrungswelt amerikanischer Collegestudenten. Für den Gebrauch an deutschen Schulen oder Hochschulen (auch Volkshochschulen) muss man die Auswahl der Beispiele und die dabei verwendeten Originalzitate und Datensätze anpassen. Das erfordert erhebliches Engagement. Wenn man aber gleichzeitig den Mut hat, aktives Üben in wirklich nennenswertem Umfang einzufordern, bietet der Ansatz und die Themenauswahl von *Common Sense Mathematics* die Chance, Leuten die Angst vor Zahlen zu nehmen und ihnen zu helfen, präsentierte Zahlen kritisch zu hinterfragen.

